# A panel of two miRNAs correlated to systolic blood pressure is a good diagnostic indicator for stroke

**DOI:** 10.1042/BSR20203458

**Published:** 2021-01-12

**Authors:** Yujun Qi, Mingfeng Yuan, Qiong Yi, Yan Wang, Lei Xu, Changsong Xu, Min Lu

**Affiliations:** 1 Department of Rehabilitation Medicine, The Affiliated Huai’an No.1 People’s Hospital of Nanjing Medical University, China; 2 Shuyang Minghe Rehabilitation Hospital, Shuyang County, Jiangsu Province, China; 3 Department of Neurology, The Affiliated Huai’an No.1 People’s Hospital of Nanjing Medical University, China

**Keywords:** bioinformatics, diagnostic indicator, serum miRNAs, stroke, WGCNA

## Abstract

**Background:** We aimed to develop a diagnostic indicator of stroke based on serum miRNAs correlated to systolic blood pressure.

**Methods:** Using miRNA expression profiles in GSE117604 from the Gene Expression Omnibus (GEO), we utilized the WGCNA to identify hub miRNAs correlated to systolic blood pressure (SBP). Differential analysis was applied to highlight hub differentially expressed miRNAs (DE-miRNAs), whereby we built a miRNA-based diagnostic indicator for stroke using bootstrap ranking Least Absolute Shrinkage and Selection Operator (LASSO) regression with 10-fold cross-validation. The classification value of the indicator was validated with receiver operating characteristic (ROC) analysis in both the training set and test set, as well as quantitative real-time PCR (qRT-PCR) for the feature miRNAs. Further, target genes of hub miRNAs and hub DE-miRNAs were retrieved for functional enrichment.

**Results:** A total of 447 hub miRNAs in the blue modules were significantly correlated with systolic blood pressure (*r* = 0.32, false discovery rate = 10^−6^). Target genes predicted with the hub miRNAs were mostly implicated in the Kyoto Encyclopedia of Genes and Genomes (KEGG) terms including mitogen-activated protein kinase (MAPK) pathway, senescence, and TGF-β signaling pathway. The diagnostic indicator with miR-4420 and miR-6793-5p showed remarkable performance in the training set (area under curve [AUC]= 0.953), as well as in the test set (AUC = 0.894). Results of qRT-PCR validated the diagnostic value of the two miRNAs embedded in the proposed indicator.

**Conclusions:** We developed a panel of two miRNAs, which is a good diagnostic indicator for stroke. These results require further investigation.

## Introduction

Stroke, also known as cerebral vascular accident, is one of the most prevalent causes of death and long-term disability worldwide [1,2]. A comprehensive understanding of risk factors may contribute to intervention to reduce the risk of stroke, identification of diagnostic biomarkers, as well as a better chance of functional recovery. It has been well-established that hypertension is the most prominent risk factor of stroke independent of stroke subtypes (hemorrhagic or ischemic) [3]. With its modifiable nature, blood pressure holds potential in the identification of diagnostic and predictive biomarkers for stroke. Surrogate biomarkers could be developed using transcriptomic data correlated to blood pressure, including serum mRNA and non-coding RNA expressions. Among them, microRNAs (miRNAs) have shown clinical relevance to diagnosis, prognosis, and therapy in the context of stroke [4].

MiRNAs, a group of small non-coding RNAs, bind to the 3′ untranslated regions of target mRNAs and play a suppressive role in post-transcriptional regulation of gene expression [5,6]. Specifically, miRNAs could be exploited for noninvasive detection from samples of body fluids such as peripheral blood [7]. In this context, clinical implications of serum miRNAs have been reported in a variety of diseases including stroke [8–11]. Researches have shown signatures based on serum miRNAs could predict the risk of stroke [12], and hold promise to be prognostic biomarkers in survivors with neurological neoplasms [13,14]. Besides, miRNAs are implicated in various biological processes including immune response [15], regulation of cell cycles [16], and cellular metabolism [17]. As such, we hypothesized that serum miRNAs correlated to systolic blood pressure (SBP) could be diagnostic biomarkers for stroke.

Using miRNA profiles from the Gene Expression Omnibus (GEO) database, we identified miRNAs correlated to SBP via Weighted Gene Co-expression Network Analysis (WGCNA) [18,19], and built a diagnostic indicator with two miRNAs by integrated analysis. Meanwhile, functional implications of featured miRNAs, as well as downstream mRNAs, were explored with bioinformatics methods.

## Materials and methods

### Flowchart and data source

**Figure 1:**
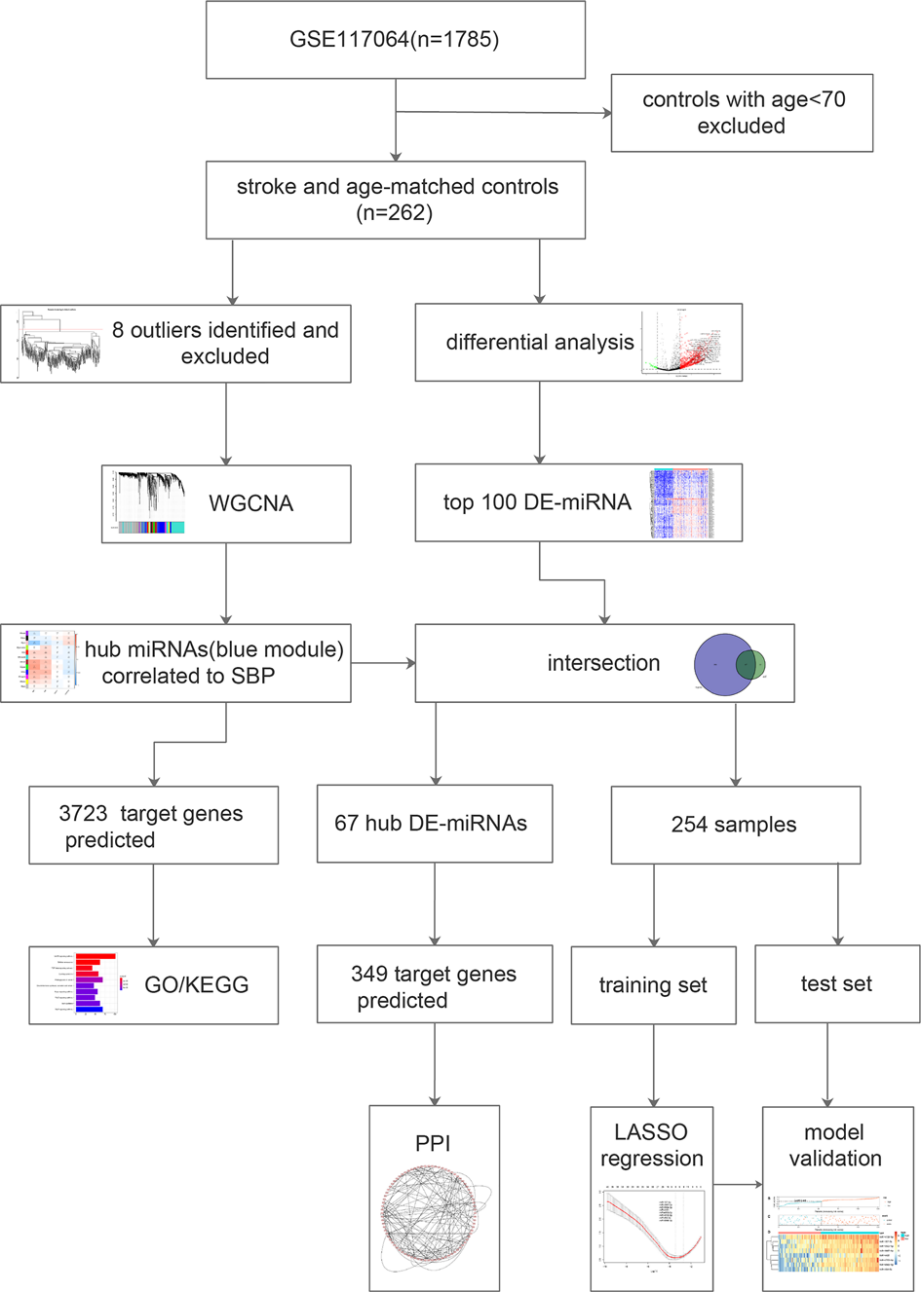
Workflow of the overall analysis

The workflow of the overall analysis is presented in Figure 1. From the GEO database (https://www.ncbi.nlm.nih.gov/geo/), the miRNA expression matrix of GSE117064 was obtained [12]. The dataset embedded a total of 1785 serum samples, out of which 262 samples were collected from individuals with age >69 years, comprising 173 samples of stroke patients and 89 samples of age-matched controls. Analyses of the present study were performed using the 262 samples.

### WGCNA and functional enrichment analysis

The weighted gene correlation network analysis (WGCNA) [18,19] was performed to construct a co-expression network in an effort to identify hub miRNAs correlated to clinical features using the ‘WGCNA’ package. Samples and miRNAs were filtered with good sample test and good gene test, while outliers of samples were further detected with hierarchical clustering. Filtered miRNAs were used to construct a scale-free network by calculating the connection strength between miRNAs. We assessed the correlation among miRNA modules, as well as their correlations to clinical traits. To narrow down the candidate miRNAs, we specifically focused on the miRNA module significantly associated with systolic blood pressure in elderly individuals. *P* values were adjusted with the false discovery rate (FDR) method [20], where FDR < 0.05 and correlation coefficient (*r*) > 0.3 were considered statistically significant. The potential target genes of selected hub miRNAs embedded in the module were predicted using *FunRich* software 3.1.3 [21]. The Gene Ontology (GO) terms and the Kyoto Encyclopedia of Genes and Genomes (KEGG) pathways enrichment analysis via the *clusterProfiler* R package [22].

### Differential analysis and identification of hub DE-miRNAs

Differentially expressed miRNAs (DE-miRNAs) between stroke and control samples using the Linear Models for Microarray Data (LIMMA) package [23] in R software, with the *P* values adjusted via the FDR as well [20]. The threshold for identification of DE-miRNAs was FDR < 0.05 and |log_2_ FC| > 1. Expression of the top 100 DE-miRNAs with the most significant FDR values were presented with a heatmap and volcano plot. Subsequently, hub DE-miRNAs were identified with the intersection of hub miRNAs identified via WGCNA and the top 100 DE-miRNAs. To identify downstream hub genes of the hub DE-miRNAs, target genes were predicted using the aforementioned method. Then, the protein–protein interaction (PPI) network was constructed via the Search Tool for the Retrieval of Interacting Genes [24] (STRING, version 10.5, https://www.string-db.org/). Genes with no less than 8 edges were considered as hub genes. Samples were also filtered with the intersection of samples in the WGCNA and differential analysis, in order to build a diagnostic indicator in downstream analyses.

### Development and validation of the diagnostic indicator

Filtered samples were randomly assigned in a 1:1 ratio to a training set and a test set, with the same proportion of stroke patients in both datasets. From the hub DE-miRNAs, we selected specific hub DE-miRNAs in the training set via the least absolute shrinkage and selection operator (LASSO) regression analysis using a bootstrap ranking procedure [25]. A 10-fold cross-validation was conducted with five external loops for intersection operation and 100 internal loops for bootstrap resampling [26,27]. Subsequently, we calculated the individualized riskscore with coefficients-weighted expression levels of specific feature DE-miRNAs and constructed a diagnostic indicator. The cutoff score was determined by the Youden method [28]. Samples with riskscores higher than the cutoff value were classified as stroke cases, while those with lower scores was classified into healthy controls. Using data in the training set, the receiver operating characteristic (ROC) analysis was performed with the area under the curve (AUC) calculated to evaluate the diagnostic accuracy. The AUC ranges from 0 to 1, with 0.7 being acceptable performance and 0.9 being excellent [29]. Besides, the diagnostic indicator was presented with risk plots incorporating the distribution of riskscores, a discriminative plot of the indicator-based classification, and heatmap of expression profiles of included hub DE-miRNAs between risk groups as defined by the indicator. Using the same formula, we assigned riskscores to individual subjects in the test set and classified them with the same cutoff value. Subsequently, the diagnostic accuracy was validated in the test set with ROC analysis and a risk plot for visualization. To detect potential overfitting of the diagnostic model, Kolmogorov–Smirnov (K-S) test based on the empirical distribution function (ECDF) was performed to compare the distributions of model outputs between the training set and the test set [30,31]. If model outputs of both datasets follow different distributions (*P*<0.05), then the model is considered overfitting [30–32]. Model outputs were calculated using the logistic function [33]: probability = *e^riskscore^*/1+*e^riskscore^* .

### Sample collection, RNA extraction, and quantitative real-time PCR (qRT-PCR)

From Shuyang Minghe Rehabilitation Hospital, we collected 10 samples with 10 ml peripheral blood in ethylenediaminetetraacetic acid (EDTA) tubes (five stroke vs. five age-match healthy controls). Blood samples were taken in 10 ml serum separator tubes (Vacusera) and centrifuged at 2000 ***g*** for 10 min to isolate the serum, from which total RNAs were extracted with MolPure Blood RNA Kit (YEASEN Biotech Co., Ltd, Shanghai, China) and reversely transcribed into complementary DNAs according to the protocol of the Goldenstar™ RT6 cDNA Synthesis Kit (Beijing TsingKe Biotech Co., Ltd, Beijing, China). SYBR Green PCR Master Mix (TsingKe) was then used for qRT-PCR analysis on Step-One Plus System (Applied Biosystems, Foster City, CA, U.S.A.). The miRNA sequences were reversely transcribed using reverse transcription primers and then amplified by upstream and downstream primers [34,35]. The primer sequences are shown in Supplementary File S1. All reactions were conducted in triplicate, and the relative expression of miRNAs was calculated by 2^−ΔΔCT^ method, with U6 small nuclear RNA (snRNA) being the internal control. The experiment was approved by the Ethics Committee of Nanjing Medical University, and all subjects provided informed consent.

### Construction of a diagnostic nomogram

With the whole dataset, a diagnostic nomogram incorporating the indicator-based miRNAs was constructed using the rms R package [36], so as to intuitively detect the risk of stroke using the miRNA panel. Additionally, decision curve analysis (DCA) [37] was conducted to investigate the clinical utility of the nomogram by evaluating the net benefits at different threshold probabilities.

## Results

### Construction of a miRNA co-expression network

**Figure 2:**
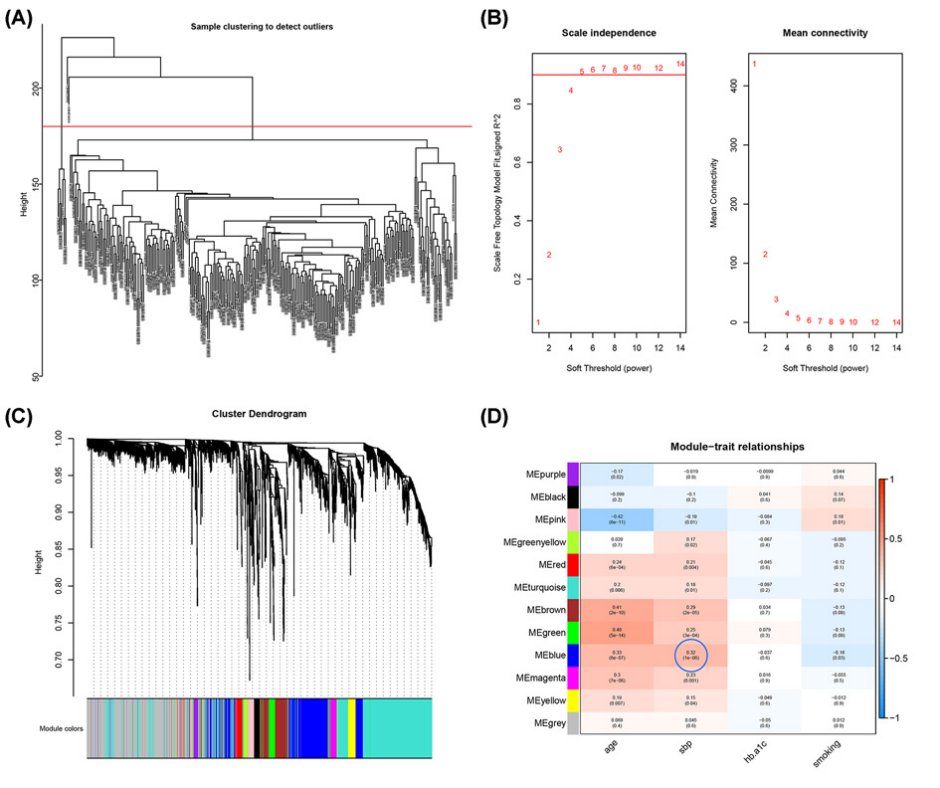
WGCNA identified hub miRNAs associated with clinical features (**A**) Clustering analysis of samples to detect outliers. (**B**) Soft threshold determination of the WGCNA. (**C**) Hierarchical clustering dendrograms of identified modules with co-expressed miRNAs. (**D**) Correlation matrix between miRNA modules and clinical traits. The number in each cell represents coefficient (*r*) indicating the strength of correlation between module and corresponding clinical trait, while the number in the parenthesis shows the *P*-value of the correlation test. The blue circle indicated miRNAs in the blue module were significantly correlated to systolic blood pressure.

**Figure 3:**
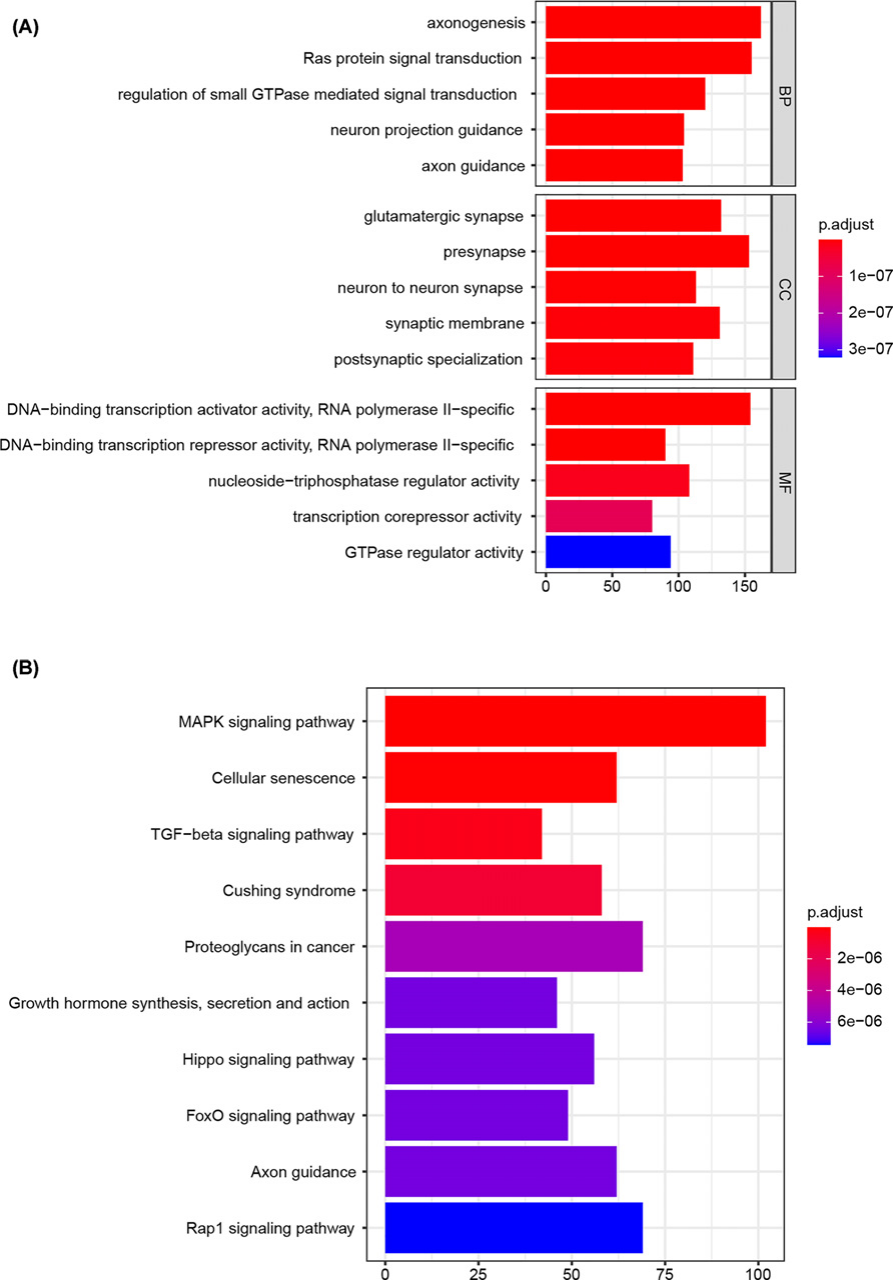
Functional enrichment analysis (**A**) The top 5 most enriched Gene Ontology (GO) terms. (**B**) The top 10 most enriched Kyoto Encyclopedia of Genes and Genomes (KEGG) pathway.

The hierarchical clustering of the samples was performed and eight outliers in samples were removed (Figure 2A). The soft-thresholding power was set at 5 with the cutoff score of scale-free *R*^2^ being 0.9 (Figure 2B). The clustering dendrograms of the sample revealed a total of 12 modules (Figure 2C), and these modules were correlated with clinical features, as presented in the heatmap plot (Figure 2D). Specifically, a total of 447 miRNAs in the blue modules (Supplementary File S2) were significantly correlated with systolic blood pressure (*r* = 0.32, FDR = 10^−6^). Hence, these miRNAs were regarded as the hub miRNAs. Target genes (Supplementary File S3) predicted with the hub miRNAs, as showed in GO enrichment, were mostly implicated in axonogenesis, glutamatergic synapse, as well as in RNA polymerase II-specific DNA-binding transcription activator activity (Figure 3A). KEGG analysis revealed the most enriched pathways including mitogen-activated protein kinase (MAPK) pathway, senescence, and TGF-beta signaling pathway (Figure 3B).

### Differential analysis and identification of hub DE-miRNAs

**Figure 4:**
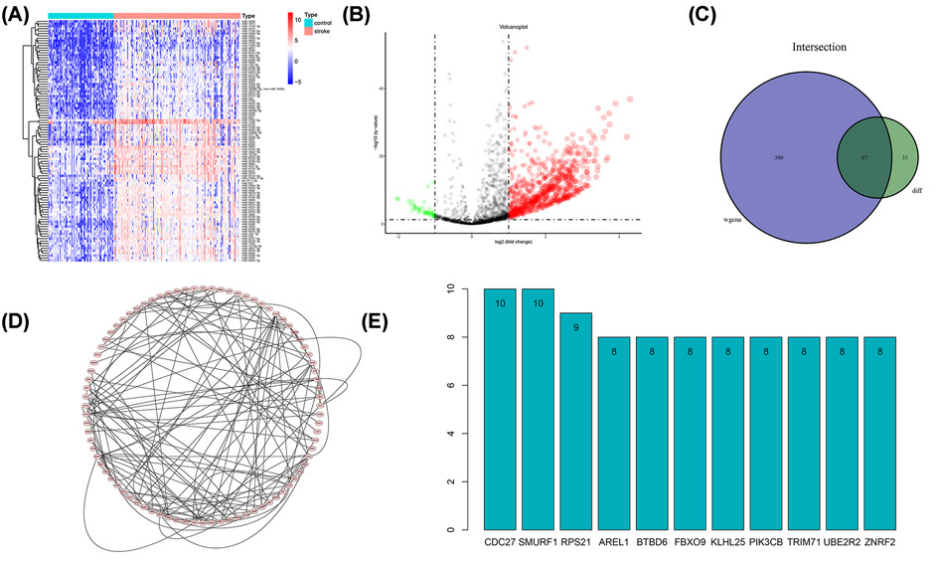
Differentially expressed microRNAs (DE-miRNAs) (**A**) Heatmap of top 100 DE-miRNAs. (**B**) Volcano plot. (**C**) Identification of hub DE-miRNAs with intersection of hub miRNAs and DE-miRNAs. (**D**) Protein–protein Interaction (PPI) network analysis of downstream genes regulated by hub DE-miRNAs. (**E**) Hub genes revealed by PPI.

Results of the differential analysis were showed in the heatmap (Figure 4A) with the top 100 DE-miRNAs, along with a volcano plot (Figure 4B). The intersection between the top 100 DE-miRNAs and hub miRNAs identified with WGCNA yielded a total of 254 serum samples and 67 hub DE-miRNAs (Figure 4C). The list of the 67 hub DE-miRNAs can be accessed in Supplementary File S4. Downstream genes (Supplementary File S5) of these hub DE-miRNAs were predicted and fitted into a PPI network (Figure 4D), which highlighted 11 hub genes, i.e., CDC27, SMURF1, RPS21, AREL1, BTBD6, FBXO9, KLHL25, PIK3CB, TRIM71, UBE2R2, and ZNRF2 (Figure 4E).

### Development and validation of the diagnostic indicator

**Figure 5:**
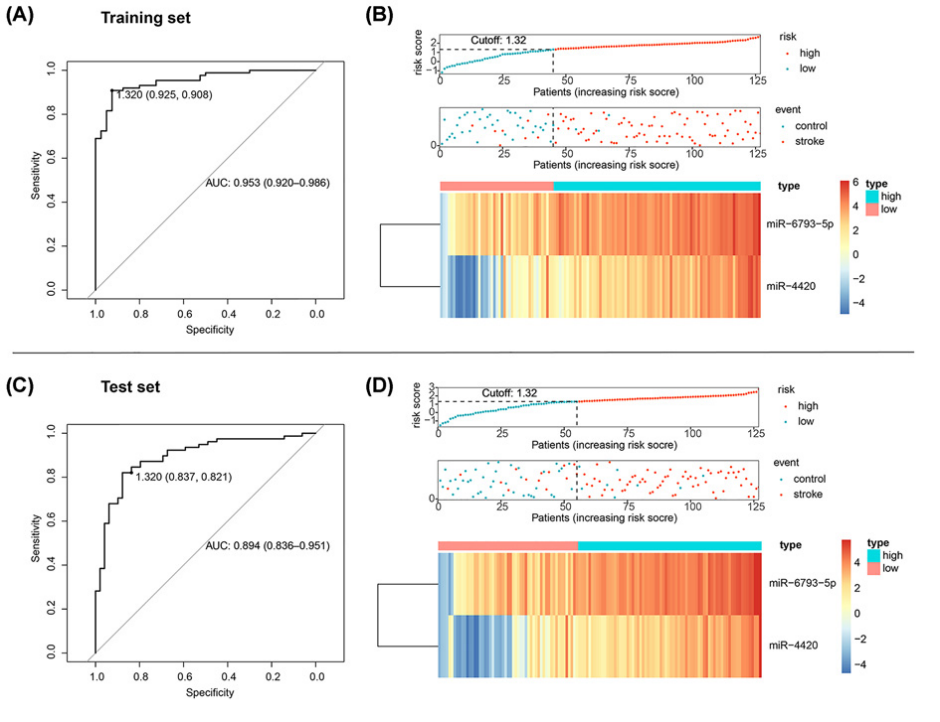
Validation of the diagnostic indicator (**A**) ROC analysis in the training set. (**B**) Risk plots for the training set. (**C**) ROC in the test set. (**D**) Risk plot for the test set. Risk plots encompass distribution of riskscores (upper panel), discriminative plot for visualization of the indicator-based classification (middle panel), and heatmap of expression profiles of included DE-miRNAs (lower panel).

**Figure 6:**
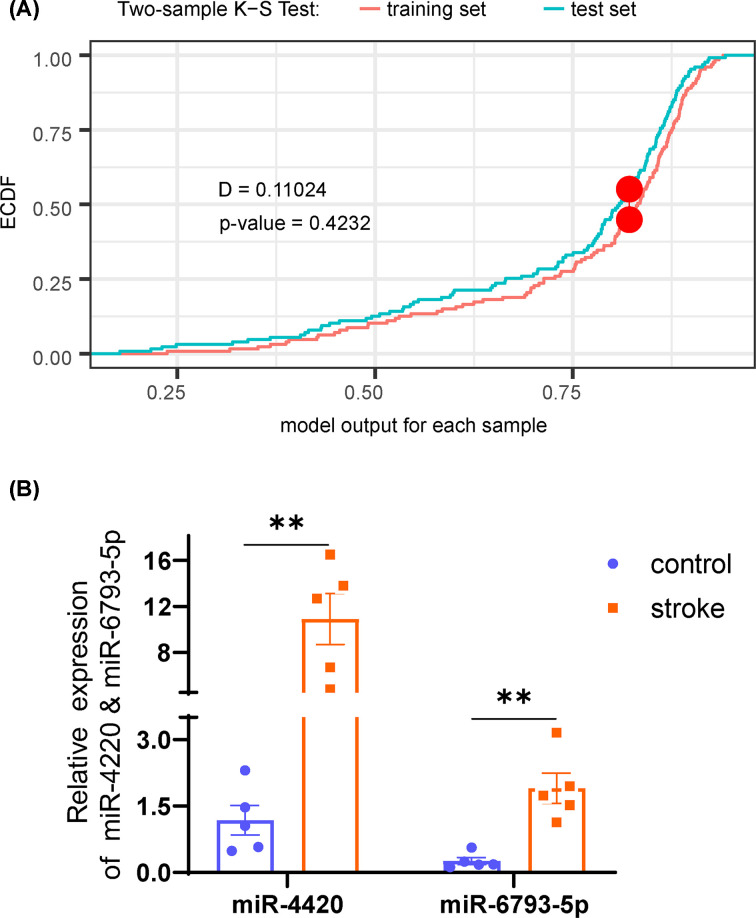
Validation of the diagnostic indicator with statistical distribution and qRT-PCR (**A**) Kolmogorov–Smirnov plot of model outputs in both the training set and test set. (**B**) Relative expression levels of miR-4420 and miR-6793-5p as evaluated by qRT-PCR. ** *P*<0.01.

Bootstrap ranking LASSO regression within the training set revealed an indicator of two hub DE-miRNAs (miR-4420 and miR-6793-5p), which yielded a diagnostic accuracy defined by AUC of 0.953 (95% confidence interval [CI]: 0.920–0.986; Figure 5A). Using the Youden method, the cutoff score was set as 1.32 with a specificity of 0.925 and a sensitivity of 0.908. As shown in the risk plot (Figure 5B), visualization of the indicator-based classification (middle panel of Figure 5B) demonstrated intuitively a highly accurate classification, while the heatmap (lower panel of Figure 5B) showed expression profiles between risk groups as defined by the indicator. To validate the robustness of the proposed indicator, the panel of two miRNAs was validated in the test set with the same formula and cutoff value using ROC analysis (Figure 5C) as well as a risk plot (Figure 5D). The diagnostic accuracy in the test set, as evaluated by the ROC curve, was 0.894 (95% CI: 0.836–0.951), while both specificity and sensitivity were higher than 0.80 at the cutoff. The risk plot (Figure 5D) demonstrated a highly accurate classification of the diagnostic indicator, as well as a similar pattern of miRNA expression. Results of K-S test demonstrated that the model outputs in the training set and test set came from the same distribution (*D* = 0.11024, *P*=0.4232), indicating no sign of overfitting (Figure 6A). Further, qRT-PCR showed distinct expression levels of miR-4420 and miR-6793-5p between stroke and control, which supports the diagnostic value of the proposed indicator (Figure 6B).

### A diagnostic nomogram with the indicator-based miRNAs and DCA

**Figure 7:**
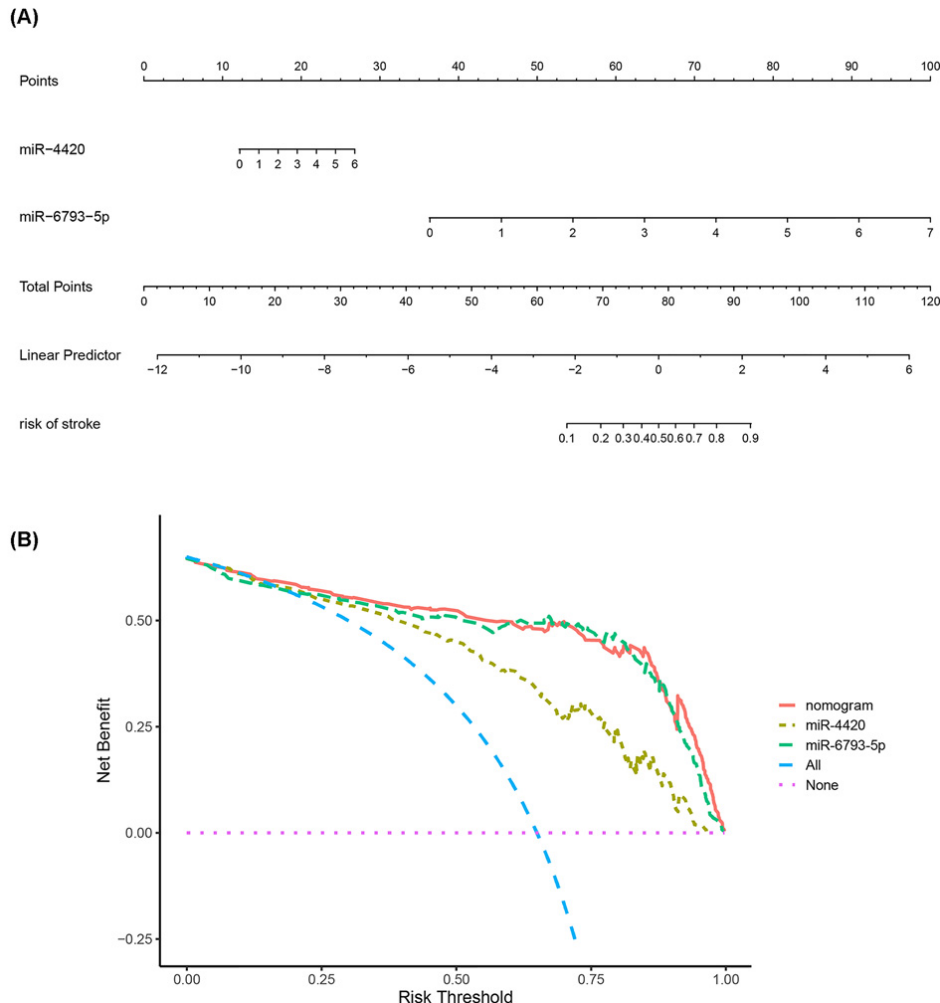
Development and validation of a diagnostic nomogram (**A**) Nomogram with expression of two indicator-based miRNAs. (**B**) Decision curve analysis

A diagnostic nomogram with the indicator-based miRNAs was presented in Figure 7A to detect the risk of stroke. Each of the two miRNAs corresponds vertically to the points, of which the sum was total points. The total points of a specific individual correspond to the risk of stroke. DCA (Figure 7B) showed that most of the red curve was above the other dashed curves, indicating net benefits of the nomogram as compared with either of miRNA, treat-none, and treat-all scheme. In other words, the clinical decisions made upon the nomogram would be favorable than other diagnostic schemes.

## Discussion

The present study utilized the WGCNA to identify hub miRNAs in the blue module correlated to SBP and applied differential analysis to highlight hub DE-miRNAs, whereby we built a robust 2-miRNA indicator for the diagnosis of stroke with remarkable classification accuracy. Results of qRT-PCR validated the diagnostic value of the two miRNAs (miR-4420 and miR-6793-5p). A diagnostic nomogram was constructed to intuitively predict the individual risk of stroke, while DCA showed a positive net benefit of the nomogram-based decisions.

The diagnostic indicator revealed two miRNAs (miR-4420 and miR-6793-5p), both of which were first reported in the present study. Target genes of hub miRNAs and hub DE-miRNAs were retrieved for functional enrichment and PPI analysis, respectively. As shown in KEGG pathway analysis, downstream genes of hub miRNAs were significantly enriched in the MAPK signaling pathway, senescence, and transforming growth factor-β (TGF-β) signaling pathway. Extensive literature indicates that the MAPK pathway was crucial in the regulation of inflammatory responses, cytokines, cell apoptosis in brain ischemia, and hemorrhage [38–40]. As such, therapies targeting the MAPK pathway could exert potentials in hypertension-related stroke. Senescence on cellular and individual levels contributes to high blood pressure, which in turn accelerates aging process and forms a vicious circle responsible for complications like stroke [41,42]. This enriched pathway, in part, explains the underlying mechanism on the interplay of aging, blood pressure, and stroke, as subjects in the present study were the elderly with age>69. TGF-β, a cytokine with multiple functions, regulates inflammatory response following injury in the brain [43]. Meanwhile, TGF-β activated kinase 1 (TAK1) correlates with the aggravation of injury [44], and contributes to an inferior long-term stroke outcome via microglial and macrophage responses [45]. Additionally, PPI with target genes of 67 hub DE-miRNAs emphasized eleven hub genes, i.e., CDC27, SMURF1, RPS21, AREL1, BTBD6, FBXO9, KLHL25, PIK3CB, TRIM71, UBE2R2, and ZNRF2. The functions of these genes in the pathogenesis of stroke warrant validation in experimental studies.

Although accumulating studies of miRNAs in stroke have been reported [4], a few studies focused on the diagnostic value of serum circulating miRNAs in stroke patients [12,46,47]. In a previous cross-sectional study, Yang et al [46] identified three circulating miRNAs (miR-107, miR-128b, and miR-153) with diagnostic accuracy of 0.97, 0.903, and 0.893 respectively. Likewise, Wang et al [47] demonstrated elevated miR-106b-5P and miR-4306, as well as decreased miR-320e and miR-320d in patients with acute stroke, showing diagnostic accuracy as high as 0.999. However, these results were subject to overfitting due to a lack of cross-validation and bootstrap resampling [48]. The diagnostic indicator in our study, albeit not as seemingly accurate as of the two aforementioned studies, was observed to be accurate and robust in the training set with 10-fold bootstrap ranking LASSO regression, as well as in the test set. Further, K-S test on model outputs detected no sign of overfitting. Similarly, a large-scale study in Japan [12] built a 3-miRNA indicator (miR-1268b, miR-4433b-3p, and miR-6803-5p) to predict the risk of stroke, which showed good predictive performance. However, the cutoff value in the training set and test set were not consistently determined, which stymies the clinical application of the miRNA indicator. In contrast, the proposed diagnostic indicator in the present study was validated in both datasets with identical cutoff value, which yielded a superior accuracy as evaluated by ROC in both datasets. Experimental validation with qRT-PCR also validated the diagnostic value of the proposed indicator. In addition, we explored the functional implications of the featured miRNAs along with target genes, which were not reported in previous studies. These findings may shed light on the design of future experiments. Of note, hub DE-miRNAs embedded in the proposed indicator did not overlap with the previous study [12], as a different modeling strategy was adopted (Figure 1).

To the authors’ best knowledge, the present study is the first report of a diagnostic indicator for stroke derived from serum miRNAs correlated to systolic blood pressure. Considering image-based techniques (computer tomography [CT] or Magnetic Resonance Imaging [MRI]) are costly, inconvenient, and unavailable in poor areas, the 2-miRNA diagnostic panel holds clinical relevance in the detection of stroke. However, the present study is subject to a few limitations. First, due to the fact that the subjects included were diagnosed with stroke without details of subtypes, subgroup analysis could not be performed. Further, the lack of function experiments in animal models is considered a major limitation. Therefore, the diagnostic performance and functional roles of these miRNAs require further investigation.

## Data Availability

The miRNA expression profile was downloaded from the GEO database (https://www.ncbi.nlm.nih.gov/geo/) with the accession number GSE117064.

## Abbreviations

AUC: area under curve
DE-miRNA: differentially expressed miRNA
GEO: Gene Expression Omnibus
KEGG: Kyoto Encyclopedia of Genes and Genomes
LASSO: Least Absolute Shrinkage and Selection Operator
MAPK: mitogen-activated protein kinase
PPI: protein–protein interaction
qRT-PCR: quantitative real-time PCR
ROC: receiver operating characteristic
SBP: systolic blood pressure
